# Spatiotemporal variations and influencing factors of highly pathogenic avian influenza risk in the Pan-Tibetan Plateau region

**DOI:** 10.1186/s40249-026-01506-w

**Published:** 2026-11-02

**Authors:** Hongyan Ren, Yao Wang, Runhe Shi

**Affiliations:** 1 State Key Laboratory of Resources and Environmental Information System, Institute of Geographic Sciences and Natural Resources Research, Chinese Academy of Sciences, Beijing, 100101 China; 2 Key Laboratory of Geographic Information Science, Ministry of Education, East China Normal University, Shanghai, 200241 China; 3 School of Geographic Sciences, East China Normal University, Shanghai, 200241 China

**Keywords:** Highly pathogenic avian influenza, Pan-Tibetan Plateau, Wild bird, Domestic poultry, Risk mapping

## Abstract

**Background:**

Highly pathogenic avian influenza (HPAI) is a major infectious disease threatening the poultry industry and public health severely. The spatiotemporal patterns and driving forces, however, remain elusive in the Tibetan Plateau and its vicinity, an ecologically critical corridor for migratory birds. This study aims to identify the factors driving these variations and to assess the HPAI risk in wild birds and domestic poultry across this region.

**Methods:**

Based on HPAI records from the Food and Agriculture Organization of the United Nations and the Official Veterinary Bulletin and 34 potential drivers in the Pan-Tibetan Plateau (PTP), comprising Xizang Autonomous Region (XZ), Qinghai Province (QH), Gansu Province (GS), Sichuan Province (SC), Yunnan Province (YN) and Xinjiang Uygur Autonomous Region (XJ), this study utilized the MaxEnt models to simulate spatial patterns of HPAI risk of wild birds, domestic poultry, and the integration of both, and identify key risk factors.

**Results:**

Performances of models for wild birds and domestic poultry models were robust (both Test AUC values > 0.9). Although the mixed model performed acceptably overall, it significantly underestimated the infection risk in wild birds. High-risk areas of wild birds HPAI were concentrated in XZ, QH, GS and XJ (> 93%), showing a close relationship with natural factors like slope and distance to water sources. Conversely, those for domestic poultry were mainly distributed in YN, SC, GS (about 70%) and the peripheries of provincial capitals, dominated by socioeconomic variables such as gross domestic product and road networks. Moreover, overlapping HPAI risk areas were found in GS, XJ, XZ, and QH, which may be associated with increasing poultry production and fragmented wetland landscape. Additionally, it’s recommended that HPAI risk modeling be stratified by host types, particularly in areas with sparse surveillance data.

**Conclusions:**

HPAI risks in wild birds and domestic poultry are dominated by natural factors and socioeconomic conditions respectively, and overlapping high-risk areas require focused attention. This study contributes to the understanding of HPAI transmission mechanisms in different hosts, providing a scientific basis for differentiated surveillance in the PTP.

**Graphical Abstract:**

**Figure:**
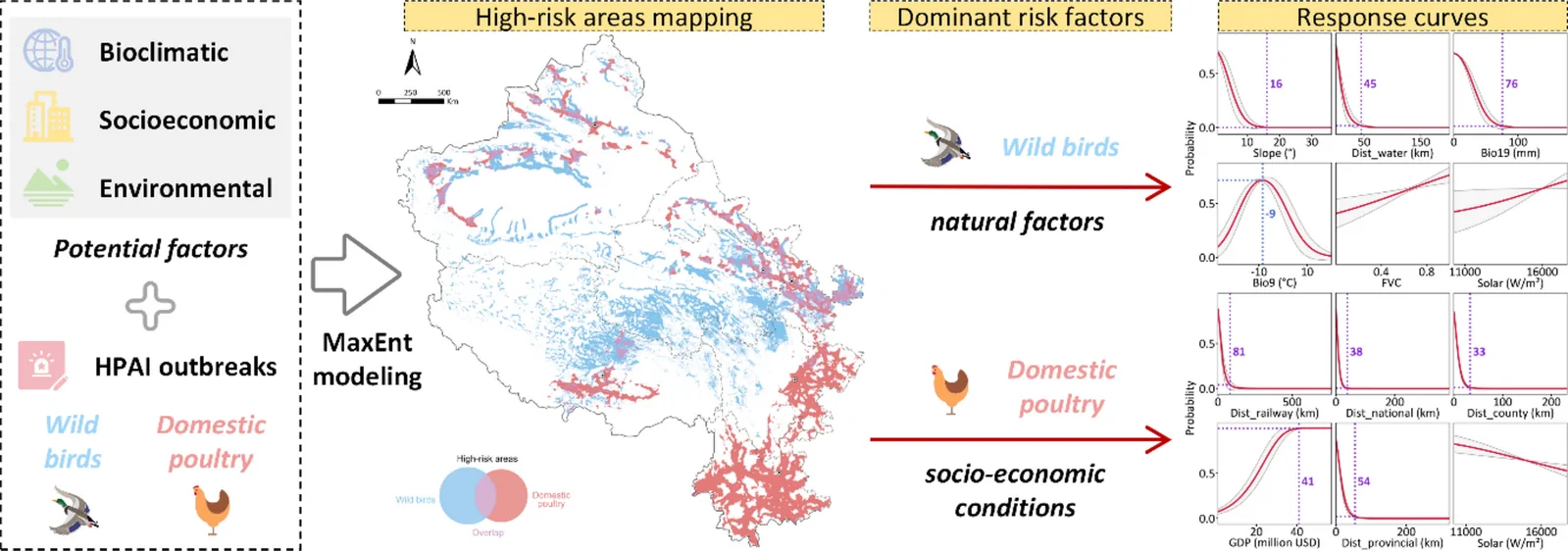

**Supplementary Information:**

The online version contains supplementary material available at https://doi.org/10.1186/s40249-026-01506-w.

## Background

Highly pathogenic avian influenza (HPAI) has become a major threat to global public health due to rapid viral evolution, worldwide prevalence and high mortality rates [1–3]. The global spread of HPAI viruses is mainly attributed to the long-distance migration of wild birds, the expansion of domestic poultry trade, as well as ongoing mutation and silent circulation among multiple hosts [4–7]. However, risk of HPAI infection in China and its potential determinants remain poorly understood, especially on the Tibetan Plateau and its vicinity.

The large-scale, cross-species transmission of avian influenza viruses depends largely on environmental factors and socioeconomic conditions. As natural hosts of avian influenza viruses, wild birds tend to carry the viruses to their habitats or stopovers along the migration routes [8–10], which are often affected by climate change and human activities [11–13]. In addition, viruses are probably transmitted from wild birds to domestic poultry, particularly when wild birds inhabit or stop over in areas close to the zones of intense human activities [6, 12]. The spread and transmission of avian influenza viruses may be accelerated by increasingly developed poultry trade networks [14].

HPAI outbreaks have occurred in more than 25 provincial-level administrative divisions (PLADs) in China so far [15]. HPAI risk in China presents geographical heterogeneity, with wild birds being more susceptible in the north and domestic poultry in the south [16]. Factors such as open water, distance to water, and impervious surfaces are significantly associated with viral infection in wild birds [2], while vaccination, poultry trade, breeding density, and wild birds’ migration play important roles in domestic poultry epidemics [17]. Since the first large-scale occurrence of HPAI at Qinghai Lake in 2005, the Tibetan Plateau and its vicinity, collectively referred to as the Pan-Tibetan Plateau (PTP), continuously suffered from HPAI outbreaks and remained at a moderate risk level [18, 19]. According to data from the Food and Agriculture Organization of the United Nations (FAO), approximately 30% of HPAI outbreaks in wild birds and 10% of those in domestic poultry in China originated in the PTP. Furthermore, since the onset of the global epidemic in 2020, more than 50% of wild bird outbreaks in China have come from this region, indicating substantial epidemic activity in the PTP.

Previous studies of avian influenza virus transmission patterns and associated factors have improved our understanding of HPAI outbreaks in China. However, the spatiotemporal differences of HPAI risk in wild birds and domestic poultry in the PTP, as well as their relationships with regional environmental and socioeconomic factors remain inadequately elucidated.

Thus, we conducted this study to (1) characterize the spatiotemporal disparities of HPAI outbreaks from 2004 to 2024 in the PTP region; and (2) identify the potential impacts of environmental factors and socioeconomic conditions on high HPAI risk of both wild birds and domestic poultry. This research would provide useful clues for strengthening HPAI surveillance and prevention and control in the pivotal regions with migration routes of wild birds.

## Methods

### Study area

**Fig. 1:**
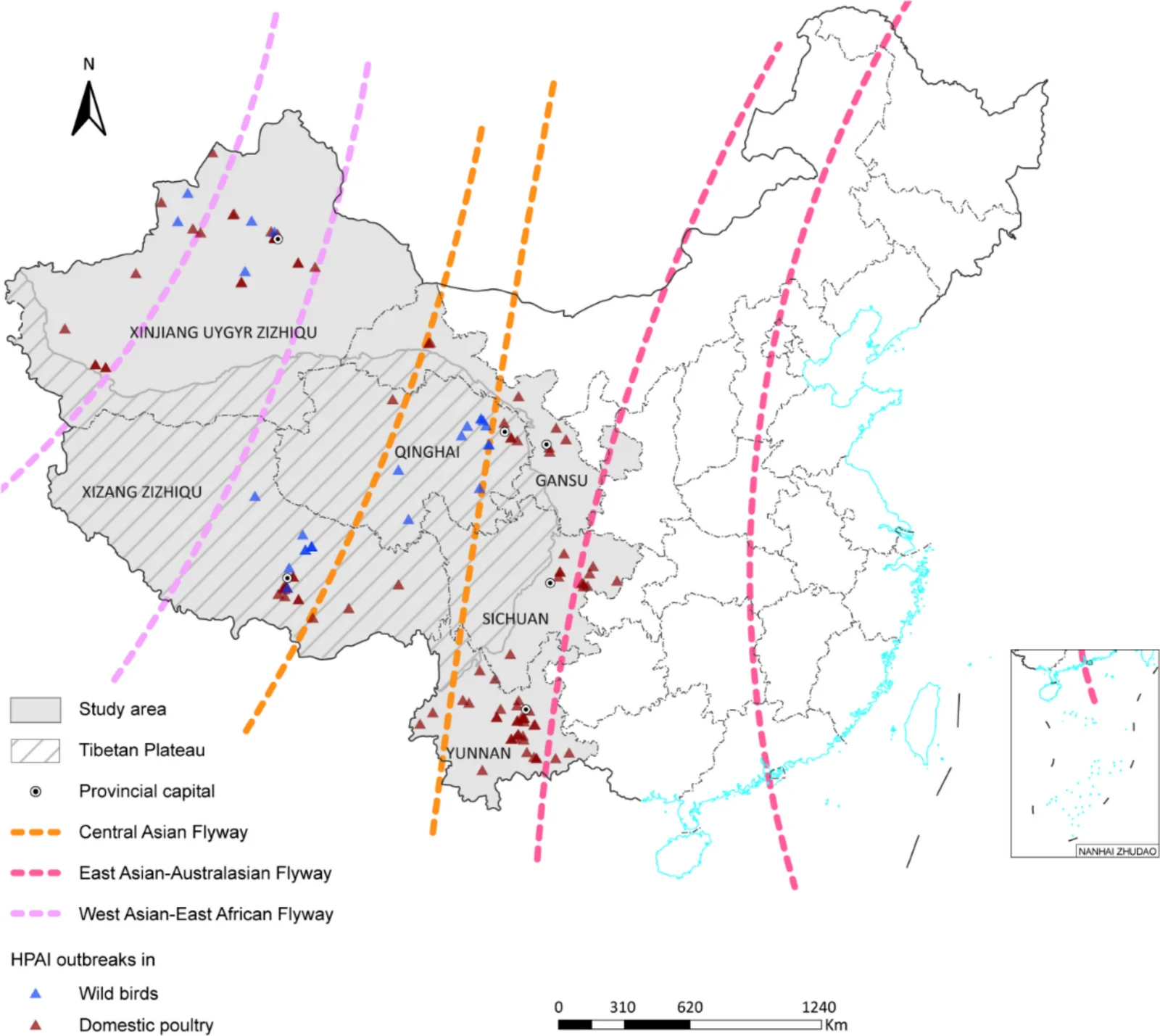
HPAI outbreaks in the PTP region and three flyways passing through. HPAI: Highly pathogenic avian influenza; PTP: Pan-Tibetan Plateau. Map approval No.: GS (2026) 4664

The PTP region is composed of Xizang Autonomous Region (XZ), Qinghai Province (QH), Gansu Province (GS), Sichuan Province (SC), Yunnan Province (YN), and Xinjiang Uygur Autonomous Region (XJ) (Fig. 1). This vast and sparsely populated region has complex and diverse climatic and geographical habitat conditions, as well as numerous lakes, rivers, and wetlands. Of the nine global migratory bird flyways, three ones, namely the West Asian-East African Flyway, Central Asian Flyway, and East Asian-Australasian Flyway, pass through this region [20, 21]. Numerous migratory birds migrate, stop over, and breed here annually, leading to a high risk of HPAI outbreaks and transmission [20, 22].

### Data collection

Data on HPAI were sourced from the FAO (https://data.fao.org/), the Official Veterinary Bulletin(https://www.moa.gov.cn/gk/sygb/), and relevant literature. A total of 137 HPAI outbreaks (26 in wild birds, 111 in domestic poultry) were collected, including location, reporting date, infected species, confirmation status, and virus subtype (H5Nx, H7N9, etc.).

According to previous research [17, 23, 24], this study selected 34 potential variables, including 19 bioclimatic, 8 socioeconomic and 7 environmental variables (Table 1). All original data were clipped to the study area and resampled to a resolution of 2 km × 2 km. The mean values of gross domestic product (GDP), population density (Pop), wind speed (Wind), downward surface shortwave radiation (Solar) and fractional vegetation cover (FVC) were further calculated based on the annual or monthly values. Descriptive statistics of variables are illustrated in Additional file 1: Table S2, Table S3.

**Table 1:** Data collection and sources used in this study

Variable group	Selected variables	Time scale	Data unit	Resolution	Sources	Wild	Domestic	Mixed
Bioclimatic variables [17]	Annual mean temperature (Bio1)	Annual	Degree Celsius	1 km	Worldclim version 2 (https://worldclim.org/data/worldclim21.html)			
	Mean diurnal range (Bio2)		Degree Celsius			√	√	√
	Isothermality (Bio3)		No unit				√	√
	Temperature seasonality (Bio4)		No unit					
	Max temperature of warmest month (Bio5)		Degree Celsius					
	Min temperature of coldest month (Bio6)		Degree Celsius					
	Temperature annual range (Bio7)		Degree Celsius					
	Mean temperature of wettest quarter (Bio8)		Degree Celsius					
	Mean temperature of driest quarter (Bio9)		Degree Celsius			√	√	√
	Mean temperature of warmest quarter (Bio10)		Degree Celsius					
	Mean temperature of coldest quarter (Bio11)		Degree Celsius					
	Annual precipitation (Bio12)		mm					
	Precipitation of wettest month (Bio13)		mm					
	Precipitation of driest month (Bio14)		mm				√	
	Precipitation seasonality (Bio15)		No unit			√	√	√
	Precipitation of wettest quarter (Bio16)		mm					
	Precipitation of driest quarter (Bio17)		mm					
	Precipitation of warmest quarter (Bio18)		mm					
	Precipitation of coldest quarter (Bio19)		mm			√		√
Socioeconomic variables [17, 23]	Gross domestic product (GDP)	Annual	Millions of 2017 USD	1 km	Gridded revised real gross domestic product [29]		√	√
	Population density (Pop)	Annual	Persons/km^2^	1 km	WorldPop (https://hub.worldpop.org/)		√	√
	Distance to railways (Dist_railway)	Annual	km	2 km	OpenStreetMap (https://www.openstreetmap.org/)		√	√
	Distance to national highways (Dist_national)						√	√
	Distance to provincial highways (Dist_provincial)						√	√
	Distance to county roads (Dist_county)						√	√
	Chicken density (Chicken)	Annual	Birds/km^2^	1 km	Gridded Livestock of the World (http://www.fao.org/livestock-systems/en/)		√	√
	Duck density (Duck)	Annual		10 km				
Environmental variables [24]	Elevation (DEM)	Annual	m	90 m	SRTM 90 m DEM (https://srtm.csi.cgiar.org/srtmdata/)			
	Slope		Degree	2 km		√		√
	Aspect		No unit	2 km		√	√	√
	Wind speed (Wind)	Annual	m/s	4 km	TerraClimate (https://climate.northwestknowledge.net/TERRACLIMATE/)	√	√	√
	Downward surface shortwave radiation (Solar)		W/m^2^			√	√	√
	Fractional vegetation cover (FVC)	Monthly	No unit	250 m	National Tibetan Plateau/Third Pole Environment Data Center (https://data.tpdc.ac.cn/)	√	√	√
	Distance to water sources (Dist_water)	Annual	km	2 km	OpenStreetMap (https://www.openstreetmap.org/)	√		√

The Wild, Domestic and Mixed respectively represented the models for wild birds, domestic poultry and the combination of both. “√” indicated that the corresponding variable was included in the model

### Risk mapping and identification of the driving forces

The MaxEnt model is established by the maximum entropy theory, and allows accurate prediction based on a small number of presence points [25], which has been widely applied in assessing the potential distribution of ecological or environment-related diseases [17, 26]. Three models were developed for wild birds, domestic poultry and the combination of both, and named as the Wild, Domestic, and Mixed, respectively.

The variance inflation factors (VIF) and Spearman’s rank correlation coefficient *ρ* of these variables were calculated to perform multicollinearity tests. Variables with VIF > 10 and |*ρ*|> 0.8 were excluded based on their ecological meanings (Additional file 1: Table S4, Fig. S2) [23, 24]. Additionally, outbreaks of wild birds are often detected near highways or within the management range of monitoring stations. This may result from the higher density of monitoring facilities in areas of human activity compared to remote areas, thus creating a false association between detection location and human activity. Therefore, socioeconomic factors were excluded from the Wild. Furthermore, considering the potential virus transmission of wild birds to domestic poultry, the simulation result was incorporated into the Domestic (variable *Wild*). The final input variables for the models are shown in Table 1.

The corresponding data on HPAI were divided into training and validation sets at a ratio of 75%:25%. To minimize the influences of potential spatial autocorrelations, only one outbreak point was retained per grid cell. In addition, during the partitioning of datasets, the selected samples were spatially as evenly distributed as possible, so as to further reduce the effects of sampling bias. MaxEnt fits a model with a series of feature classes (FC) and controls the model complexity via a regularization multiplier (RM); thus, the most appropriate model was selected by evaluating different combinations of FC and RM (more details in Additional file 1) [25, 27]. Ten replicates of Bootstrap were used to build the model. The simulation results were the average of the ten replicates, and were classified into high and low-risk levels using the “Maximum training sensitivity plus specificity cloglog threshold” [28]. Variable importance was comprehensively measured based on Percent Contribution and Permutation Importance, and the mechanisms of environmental variables were analyzed via Response Curves.

The receiver operating characteristic curve and true skill statistic (TSS) were employed to evaluate the models. Model performance was judged based on the area under the curve (AUC), with an AUC > 0.9 generally suggesting high accuracy [26]. TSS represents the success rate of fitting presence and absence data and is suitable for evaluating binary predictions. Typically, a TSS > 0.5 indicates good model fitting effects [30]. Furthermore, the validation sets were overlaid with the risk simulation results for hitting analysis to further judge the performances of the models. In this study, we utilized the *usdm*, *ENMeval* and *predicts* packages in the statistical software R 4.4.3 (R Core Team, Vienna, Austria) to construct and interpret the models.

## Results

### Spatiotemporal variations of HPAI outbreaks

**Fig. 2:**
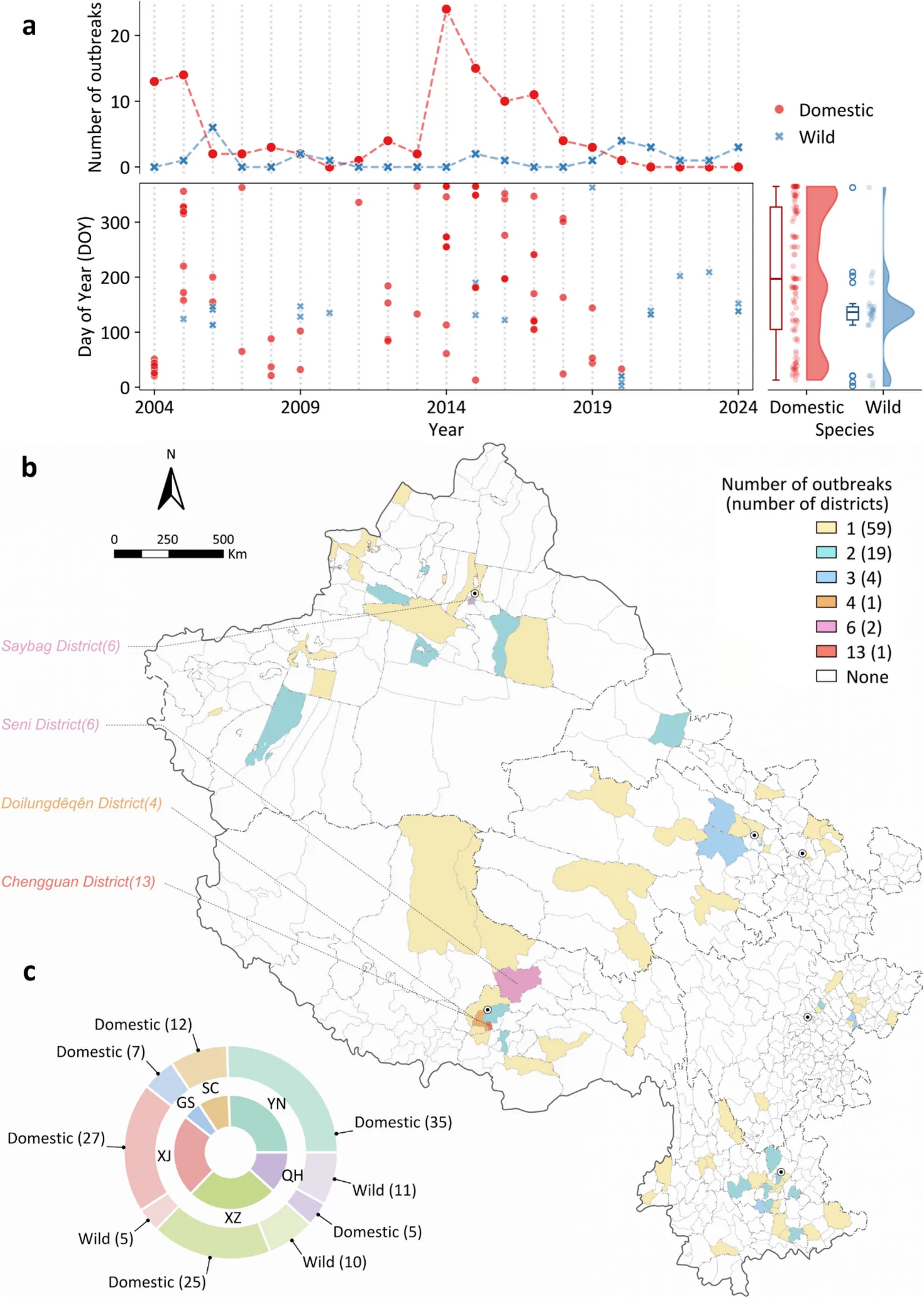
Spatiotemporal variations of HPAI outbreaks in the PTP. **a** temporal variations; **b** spatial distribution; **c** the situation of HPAI outbreaks in six provincial-level administrative divisions. HPAI: Highly pathogenic avian influenza; PTP: Pan-Tibetan Plateau; YN: Yunnan Province; GS: Gansu Province; XZ: Xizang Autonomous Region; XJ: Xinjiang Uygur Autonomous Region; SC: Sichuan Province; QH: Qinghai Province. Map approval No.: GS (2026) 4664

HPAI outbreaks in the PTP exhibited distinct temporal characteristics during 2004–2024 (Fig. 2a). Interannually, HPAI outbreaks in wild birds occurred at a low average frequency of 1.24 outbreaks/year, with higher counts in 2006 (6 outbreaks) and 2020 (4 outbreaks). By contrast, domestic poultry HPAI presented a much higher frequency (5.29 outbreaks/year), with peak frequencies during 2004–2005 (13.5 outbreaks/year) and 2014–2017 (15 outbreaks/year). Overall, the trend of HPAI outbreaks from 2004 to 2019 was dominated by domestic poultry events. Since 2021, however, HPAI outbreaks in this region have been reported only in wild birds, with no outbreaks reported in domestic poultry. In addition, regarding the day of the year (DOY) on which HPAI outbreaks were reported, wild birds’ outbreaks typically occurred during DOY 100–150, while domestic poultry outbreaks were documented in DOY 150–200 and from the 300th to the 50th day of the following year, indicating a potential temporal lag relative to wild birds.

There were 85 counties with HPAI events in the PTP with distinct spatial patterns (Fig. 2b). A total of 22 and 36 outbreaks of domestic poultry HPAI were observed in 17 YN-XJ and 23 YN-XZ counties during its two peak periods (i.e., 2004–2005, and 2014–2017), respectively. Additionally, the majority (77/85) of counties reported no more than 3 outbreaks and were dispersedly distributed, whereas several counties displayed much higher HPAI outbreaks (≥ 4), including Seni District, Saybag District, Doilungdêqên District, and Chengguan District.

These analyses showed significant spatiotemporal shifts in the predominant host type of HPAI outbreaks in the PTP, from domestic poultry during the early period to wild birds in recent years, with wild-bird outbreaks highly concentrated in counties in QH and XZ.

### Performance of models

According to the AUC values in Table 2, all three MaxEnt models performed well. The Wild and Domestic models were capable of identifying high- and low-risk areas with TSS values exceeding 0.8, and more than 95% of HPAI points in the validation set fell within high-risk areas (Additional file 1: Fig. S3). Accordingly, subsequent analyses could be conducted using these models.

**Table 2:** Evaluation of three MaxEnt models

Model	Model evaluation			Number of hits	
	Training AUC	Test AUC	TSS	Low risk	High risk
Wild	0.956 ± 0.008	0.937 ± 0.038	0.843 ± 0.035	0	7
Domestic	0.964 ± 0.008	0.953 ± 0.018	0.807 ± 0.035	1	33
Mixed	0.943 ± 0.012	0.913 ± 0.048	0.753 ± 0.041	0	40

The Wild, Domestic and Mixed respectively represented the models for wild birds, domestic poultry and the combination of both

TSS: True skill statistic; AUC: Area under the curve

### Spatial distribution of HPAI risk

**Fig. 3:**
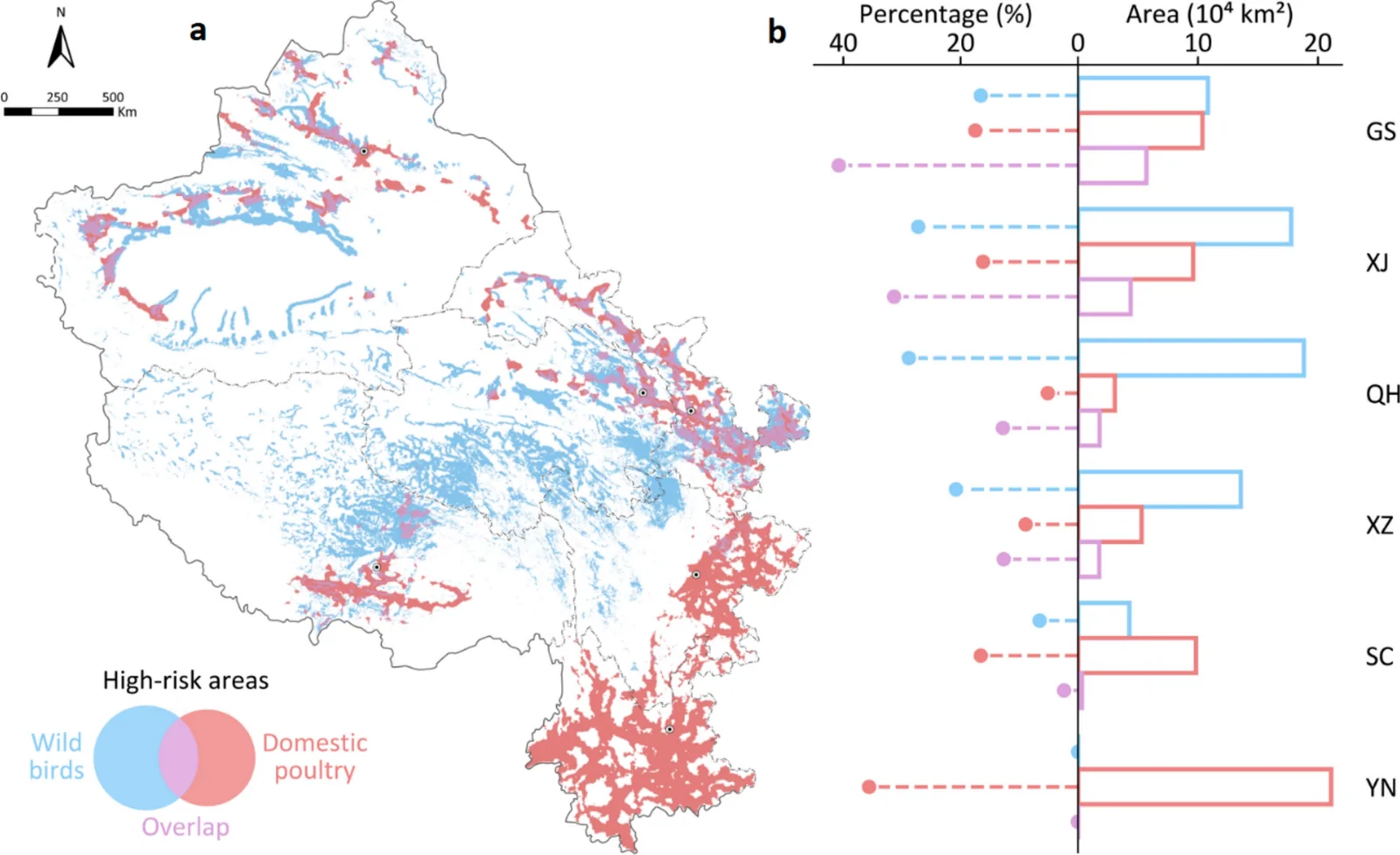
High-risk areas mapping and statistics in six provincial-level administrative divisions. **a** High-risk areas mapping and the overlap of the Wild and Domestic models; **b** high-risk areas statistics in six provincial-level administrative divisions. YN: Yunnan Province; GS: Gansu Province; XZ: Xizang Autonomous Region; XJ: Xinjiang Uygur Autonomous Region; SC: Sichuan Province; QH: Qinghai Province. Map approval No.: GS (2026) 4664

The high-risk areas derived from three models mentioned above were spatially featured across the PTP (Fig. 3). Among these models, the Mixed model exhibited spatial patterns and area proportions highly similar to those of the Domestic, which failed to fully reflect the unique high-risk areas for wild birds HPAI (Additional file 1: Fig. S4). The total high-risk area for HPAI in wild birds (652,296 km^2^) was larger than that in domestic poultry (592,208 km^2^). Within six PLADs in the PTP, QH had the largest high-risk areas and YN the smallest for wild birds HPAI. By contrast, YN accounted for the highest proportion of the total high-risk areas for HPAI in domestic poultry, whereas QH the lowest. Additionally, the high-risk areas for domestic poultry HPAI were often located on the outskirts of provincial capitals. However, some high-risk zones overlapped between HPAI in wild birds and domestic poultry, especially in four PLADs (i.e., QH, XJ, XZ, and GS).

These analyses demonstrated that the high-risk areas for HPAI in wild birds were obviously spatially differentiated from those in domestic poultry across the PTP.

### Dominant risk factors of HAPI risk

**Fig. 4:**
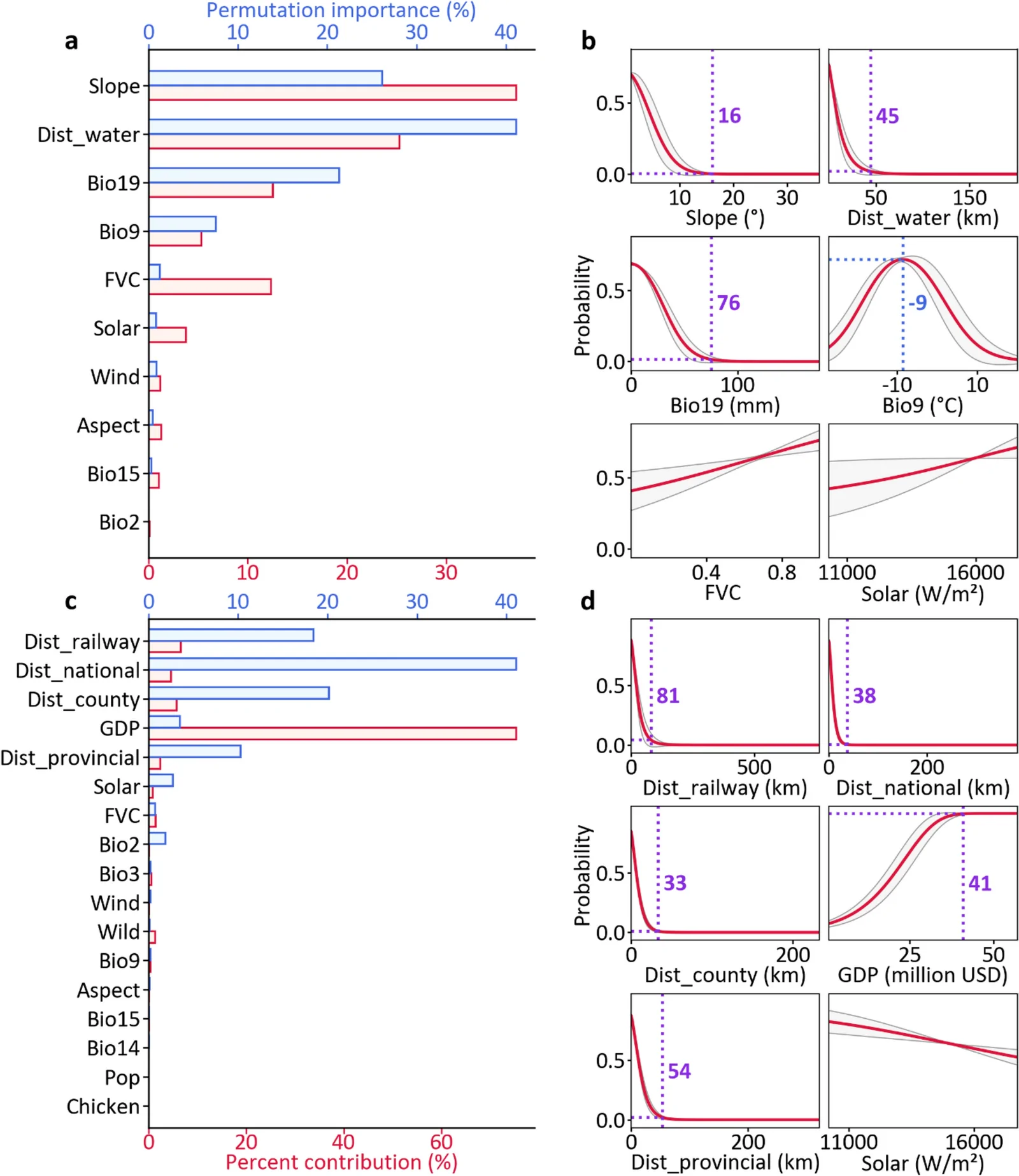
Importance of input factors based on permutation importance and percent contribution (**a**, **c**), and response curves of highly pathogenic avian influenza (HPAI) risk to dominant factors (**b**, **d**). Factors derived from **a**, **b** The Wild model; **c**, **d** The Domestic model. Bio13: Precipitation of wettest month; Bio14: Precipitation of driest month; Bio15: Precipitation seasonality; Bio19: Precipitation of coldest quarter; Bio2: Mean diurnal range; Bio3: Isothermality; Bio9: Mean temperature of driest quarter; Chicken: Chicken density; Dist_county: Distance to county roads; Dist_national: Distance to national highways; Dist_provincial: Distance to provincial highways; Dist_railway: Distance to railways; Dist_water: Distance to water sources; FVC: Fractional vegetation cover; GDP: Gross domestic product; Pop: Population density; Solar: Downward surface shortwave radiation; Wild: Simulation results of wild birds; Wind: Wind speed

According to the values of percent contribution and permutation importance, dominant risk factors identified by the Domestic model differed considerably from those of the Wild. Among the potential variables, 5 environmental factors (i.e., Slope, Dist_water, Bio19, Bio9, and FVC) posed relatively stronger impacts on the high-risk areas for HPAI in wild birds (Fig. 4a). In contrast, GDP and four distance-related variables (i.e., Dist_railway, Dist_national, Dist_county, Dist_provincial) exerted much more important effects on HPAI in domestic poultry, suggesting that socioeconomic factors dominated its distribution in this region (Fig. 4c). Similarly, dominant variables derived from the Mixed model were comparable to those from the Domestic (Additional file 1: Fig. S5).

In addition, HPAI risk comprehensively responded to above key factors, and the resulting response curves could be classified into three categories: Type 1, an initial rise or decline followed by stabilization; Type 2, a gradual rise or decline; and Type 3, non-linear changes with marked turning points.

For the Wild Model (Fig. 4b), the risk of HPAI outbreaks tended to rapidly decrease along with the increase of Type 1 variables in the zones with lower values than 16° (Slope), 45 km (Dist_water), 76 mm (Bio19). And then the risk approached zero in the areas with larger values than above thresholds. The risks often exhibited an upward or downward trend with the increase of Type 2 variables (i.e., FVC, Solar, Aspect, Bio15, and Bio2) or the decrease of Wind (wind speed) across the PTP. In the areas with temperature below − 9 °C (Bio9, the single variable in Type 3), the risks leaned to rise with increasing Bio9, whereas it turned to decline in the zones with temperature beyond —9 °C.

The risks of domestic poultry HPAI tended to gradually risewith GDP growth, especially in the regions with lower GDP values than USD 41 million (in 2017 dollars) (Fig. 4d). When GDP surpassed this threshold, the risks remained high, at approximately 1.0. In the zones with distances of 81, 38, 54 and 33 km from railways, national highways, provincial highways, and county roads, the closer to the roads, the higher the HPAI risk. The risk was apt to zero when the distances exceeded these thresholds. Dominant variables were converted into binary maps according to thresholds (Additional file 1: Fig. S6), which fell within the observed ranges in the PTP and could therefore be incorporated into risk mapping.

These results showed that natural environmental factors dominated the risk of HPAI outbreaks in wild birds, whereas socioeconomic conditions were the primary predictors of the risk in domestic poultry; the corresponding response curves for these key variables also differed markedly between the two host groups.

## Discussion

As a pivotal zone of global migration for wild birds, the PTP region plays an important role in the worldwide and China’s prevalence of HPAI. In this study, we analyzed the spatiotemporal variations of HPAI events over the past two decades, and then simulated the HPAI outbreak risks of wild birds and domestic poultry across this region, by which several notable findings were achieved. This study would provide useful clues for strengthening the surveillance and interventions on the prevalence of HPAI.

Frequent HPAI outbreaks were spatiotemporally differentiated in the PTP over the past two decades. Wild birds HPAI broke out more often in XZ, QH, and XJ, while domestic poultry HPAI seemed more likely to occur in GS, YN, and SC. This phenomenon could be explained by the differences of either wild birds’ habitats or poultry farming volume among these PLADs [16, 31]. In the PTP, HPAI outbreaks of wild birds were temporally clustered in the period of waterfowl breeding (around May), and those of domestic poultry often rose in June-July and reached a peak during the migratory season (November and December), which was consistent with existing studies [3]. In addition, there was an obvious transition of HPAI events’ compositions from the earlier co-occurrence of wild birds and domestic poultry to current dominance of wild birds, which may be reasonably explained by the implementation of a nationwide, comprehensive vaccine immunization program [32]. Accordingly, it is suggested that vigilance against HPAI in wild birds should be sustained in XZ, XJ, and QH. Concurrently, monitoring of domestic poultry should be intensified in GS, SC, and YN immediately following any outbreak of the virus in wild birds within these PLADs.

Previous studies have confirmed that the MaxEnt model does well in simulating species distribution or disease risk over large areas even with small sample sizes [26, 33]. By means of this method, our investigation simulated the HPAI risk across the PTP. The high-risk areas for wild birds HPAI were concentrated in the most areas of XZ-QH-GS and central XJ, while those for domestic poultry HPAI were densely distributed in YN, GS, eastern SC, and the peripheries of provincial capitals in XZ, QH, and XJ. In larger-scale or global studies of HPAI in domestic poultry [17, 34, 35] and wild birds [2, 12], both risk probabilities were generally high in the PTP, especially for wild birds around Qinghai Lake and domestic poultry in eastern SC, which could align well with the overall distribution pattern observed in our study.

Meanwhile, there were many zones of high HPAI risk for not only domestic poultry but also wild birds in GS, XZ, QH, and XJ, which may be closely associated with a series of key spaces facilitating the virus mutation and spillover caused by frequent co-occurrence of common wetland landscape (typical habitats of migratory birds) and increasing poultry production [12, 14]. However, the validation of the Wild Model was inevitably restricted by the sparse HPAI events data, which is a common issue in current research on HPAI risk of wild birds [2, 12]. As a result, the HPAI events of wild birds were often obviously fewer than those of domestic poultry, by which the HPAI risk of wild birds tended to be heavily underestimated in the Mixed. Therefore, we recommend that much more attention should be paid to the zones with overlapped HPAI risk in the PTP, and current wild birds HPAI monitoring system ought to be greatly improved for collecting more HPAI events data of good quality.

HPAI risk patterns of wild birds and domestic poultry tended to be shaped by different key influencing variables in this region. HPAI risk of wild birds was mainly influenced by slope and the distance to water sources, which was probably related to wild birds’ activities and habitats in flat areas near water bodies [2]. In contrast, HPAI risk of domestic poultry was subjective to complex socioeconomic factors in the zones surrounding provincial capitals due to relatively higher economic level, larger density of road networks, and more active trade of domestic poultry [4, 23]. In a word, HPAI risk of wild birds and domestic poultry were spatially differentiated in this region. And thus, some targeted interventions (e.g., regulation of live poultry trade, and vaccination coverage promotion) should be strengthened in the regions surrounding provincial capitals, and unmanned monitoring equipment could be appropriately deployed in the habitats of migratory birds on the global flyways.

Some limitations are worth noting. First, risk simulation was probably limited by unbalanced HPAI outbreak data, particularly on wild birds closely related with underreporting and bias, which would be addressed by obtaining more data in extended observation periods or regions in the future. Second, some reasonable proxy variables under the One-Health framework would be considered in the future for the replacement of several important factors including intensive farming density and live poultry markets. Finally, although spatial variations of HPAI risk have been captured by the MaxEnt models in current investigation, models capable of describing temporal changes of HPAI events would be employed to enhance the ability of identifying and predicting dynamic risks under various scenarios.

## Conclusions

Over the past two decades, HPAI outbreaks in the PTP exhibited distinct spatiotemporal characteristics, and various environmental factors and socioeconomic conditions were differentially associated with HPAI risk in wild birds and domestic poultry. This research has improved our understanding of the situation of HPAI events and their influencing factors in the pivotal zone along global wild-bird migratory flyways, providing valuable insights for local authorities to strengthen HPAI surveillance and control.

## Supplementary Information

Additional file 1. Detailed documentation of the modeling procedures. Table S1. The optimal parameter configuration for three models. Fig. S1. The distribution of China at provincial-level administrative divisions. Table S2. Descriptive statistics of variable values extracted from wild bird outbreak points. Table S3. Descriptive statistics of variable values extracted from domestic poultry outbreak points. Table S4. VIFs of the remained variables in three models. Fig. S2. Correlation coefficients of the remained variables in three models. Fig. S3. Classification of high- and low-risk areas and hit results of validation points. Fig. S4. High-risk areas statistics derived from the model for the combination of wild birds and domestic poultry. Fig. S5. Dominant influencing factors and response curves derived from the model for the combination of wild birds and domestic poultry. Fig. S6. Spatial distribution of dominant factors’ thresholds derived from models

## Data Availability

The datasets used and analyzed during the current study are available from the corresponding author on reasonable request.

## Abbreviations

PTP: Pan-Tibetan Plateau
YN: Yunnan Province
GS: Gansu Province
XZ: Xizang Autonomous Region
XJ: Xinjiang Uygur Autonomous Region
SC: Sichuan Province
QH: Qinghai Province
GDP: Gross domestic product
Pop: Population density
Dist_railway: Distance to railways
Dist_national: Distance to national highways
Dist_provincial: Distance to provincial highways
Dist_county: Distance to county roads
Chicken: Chicken density
Duck: Duck density
DEM: Elevation
Wind: Wind speed
Solar: Downward surface shortwave radiation
FVC: Fractional vegetation cover
Dist_water: Distance to water sources
TSS: True skill statistic
AUC: Area under the curve
