# Supplementary material for: Spatiotemporal variations and influencing factors of highly pathogenic avian influenza risk in the Pan-Tibetan Plateau region
Source: Infect Dis Poverty. 2026 Nov 2;15:113. doi: 10.1186/s40249-026-01506-w (PMC13726235; doi:10.1186/s40249-026-01506-w)
Supplement: Supplementary file 1 [file 40249_2026_1506_MOESM1_ESM.docx]

**Additional file 1**

Supplementary materials to: **Spatiotemporal variations and influencing factors of highly pathogenic avian influenza risk in the Pan-Qinghai-Tibet Plateau region**.

**Table of Contents**

| Page | Item |
| --- | --- |
| 2 | Detailed documentation of the modeling procedures  Table S1 The optimal parameter configuration for three models |
| 3 | Fig. S1 The distribution of China at provincial-level administrative divisions |
| 4 | Table S2 Descriptive statistics of variable values extracted from wild bird outbreak points |
| 5 | Table S3 Descriptive statistics of variable values extracted from domestic poultry outbreak points |
| 6 | Table S4 VIFs of the remained variables in three models |
| 7 | Fig. S2 Correlation coefficients of the remained variables in three models |
| 8 | Fig. S3 Classification of high- and low-risk areas and hit results of validation points |
| 9 | Fig. S4 High-risk areas statistics derived from the model for the combination of wild birds and domestic poultry |
| 10 | Fig. S5 Dominant influencing factors and response curves derived from the model for the combination of wild birds and domestic poultry |
| 11 | Fig. S6. Spatial distribution of dominant factors’ thresholds derived from models |

**Detailed documentation of the modeling procedures**

First, the Average Nearest Neighbor analysis revealed a significantly clustered distribution pattern (Nearest Neighbor Ratio = 0.36, Z = -12.90, *p* < 0.01), indicating that our occurrence records were spatially clustered. To minimize the influences of spatial autocorrelations, we overlaid a 2 km × 2 km grid across the study area and retained only one outbreak record per grid cell for model fitting. In addition, during the partitioning of data into training and validation sets, we strove to ensure that the selected samples were spatially as evenly distributed as possible, so as to further reduce the potential effects of sampling bias and allow the model to be evaluated across the full geographic extent.

Second, MaxEnt fits a model with a series of feature classes (FC) and controls the model complexity via a regularization multiplier (RM) that applies a penalty for additional constraints. Thus, we selected the most appropriate model by evaluating different combinations of FC and RM. In MaxEnt, there are five feature types: Linear (L), Quadratic (Q), Hinge (H), Product (P), and Threshold (T). In total, 34 candidate models were created by combining 17 RM settings (1.1–1.9 at intervals of 0.1 and 0.5–4 at intervals of 0.5) and 2 combinations of FC settings (L, LQ) with the *ENMeval* package in R 4.4.3 (R Core Team, Vienna, Austria). Then we selected the combination with ΔAICc = 0 (Akaike Information Criterion corrected) as the optimal parameter configuration for the model (**Table S1**). In this procedure, the spatial block method (the parameter *partitions*) was employed for cross-validation to reduce the effects of spatial autocorrelations.

**Table S1 The optimal parameter configuration for three models**

|  | Wild | Domestic | Mixed |
| --- | --- | --- | --- |
| FC | L | L | L |
| RM | 1.8 | 1.0 | 1.4 |
| **Notes:** The Wild, Domestic and Mixed respectively represented the models for wild birds, domestic poultry and the combination of both. FC: Feature class; RM: Regularization multiplier; L: Linear. | | | |

Third, to reduce the uncertainty of the MaxEnt model, 10 replicated runs of Bootstrap were used to build the model, and the final results are the average of the ten replicates. The maximum iteration and convergence threshold was set to 5000 and 1e-6 respectively. Other calculation parameters were kept as the default. The potential species distribution map predicted by the MaxEnt model was in the cloglog format. The predicted existence probabilities of the species between 0 and 1 were reclassified into high and low-risk levels via “Maximum training sensitivity plus specificity cloglog threshold”.

**Fig.S1 The distribution of China at provincial-level administrative divisions.**

(Source: <http://bzdt.ch.mnr.gov.cn/browse.html?picId=%224o28b0625501ad13015501ad2bfc2187%22>)

**Table S2 Descriptive statistics of variable values extracted from wild bird outbreak points**

| Variables | Count | Mean | Median | Std | Min | Max | Q25 | Q75 |
| --- | --- | --- | --- | --- | --- | --- | --- | --- |
| Aspect | 26 | 185.1 | 208.0 | 81.1 | 21.9 | 340.6 | 155.6 | 235.9 |
| Bio1 | 26 | 1.7 | 0.0 | 4.2 | -5.3 | 10.1 | -0.7 | 3.5 |
| Bio2 | 26 | 13.7 | 14.0 | 1.3 | 10.2 | 15.2 | 13.2 | 14.6 |
| Bio3 | 26 | 35.5 | 37.4 | 6.1 | 21.3 | 43.8 | 34.8 | 39.6 |
| Bio4 | 26 | 936.3 | 862.6 | 226.2 | 661.6 | 1495.8 | 806.3 | 913.0 |
| Bio5 | 26 | 19.3 | 16.4 | 6.5 | 11.6 | 32.5 | 15.4 | 21.5 |
| Bio6 | 26 | -19.8 | -20.5 | 2.8 | -25.6 | -10.7 | -21.0 | -19.0 |
| Bio7 | 26 | 39.2 | 37.2 | 5.3 | 32.7 | 51.4 | 36.1 | 40.2 |
| Bio8 | 26 | 12.2 | 10.0 | 5.6 | 5.0 | 23.8 | 8.8 | 14.0 |
| Bio9 | 26 | -9.3 | -9.9 | 2.8 | -15.5 | -1.3 | -10.2 | -8.8 |
| Bio10 | 26 | 12.5 | 10.0 | 6.0 | 5.0 | 25.3 | 8.8 | 14.0 |
| Bio11 | 26 | -10.5 | -11.0 | 2.8 | -16.3 | -1.3 | -11.2 | -10.6 |
| Bio12 | 26 | 355.4 | 387.0 | 116.3 | 92.5 | 578.5 | 293.0 | 427.6 |
| Bio13 | 26 | 83.2 | 89.1 | 33.1 | 21.0 | 130.8 | 70.6 | 106.9 |
| Bio14 | 26 | 2.7 | 2.5 | 2.3 | 0.0 | 10.0 | 1.0 | 3.0 |
| Bio15 | 26 | 96.6 | 105.9 | 25.5 | 35.0 | 127.3 | 98.8 | 110.1 |
| Bio16 | 26 | 222.8 | 248.2 | 86.7 | 54.8 | 348.2 | 192.6 | 283.0 |
| Bio17 | 26 | 9.2 | 9.0 | 7.1 | 1.0 | 32.0 | 4.2 | 9.0 |
| Bio18 | 26 | 221.4 | 248.2 | 89.1 | 54.8 | 348.2 | 192.0 | 283.0 |
| Bio19 | 26 | 9.6 | 9.0 | 7.3 | 2.0 | 33.0 | 4.2 | 9.8 |
| DEM | 26 | 3320.2 | 3516.0 | 1463.6 | 483.0 | 4942.0 | 2969.2 | 4512.5 |
| Dist_water | 26 | 2.6 | 2.1 | 2.3 | 0.0 | 7.7 | 0.5 | 3.7 |
| FVC | 26 | 0.6 | 0.6 | 0.2 | 0.1 | 1.0 | 0.5 | 0.8 |
| Slope | 26 | 1.4 | 1.0 | 1.3 | 0.1 | 5.3 | 0.6 | 1.8 |
| Solar | 26 | 16127.5 | 16153.6 | 710.2 | 14891.5 | 17532.2 | 15597.6 | 16709.9 |
| Wind | 26 | 2.8 | 2.9 | 0.4 | 2.1 | 3.5 | 2.5 | 3.0 |
| **Notes:** Std: standard deviation; Min/Max: the minimum/maximum value; Q25/Q75: 25^th^ and 75^th^ percentiles. Bio1: Annual mean temperature; Bio2: Mean diurnal range; Bio3: Isothermality; Bio4: Temperature seasonality; Bio5: Max temperature of warmest month; Bio6: Min temperature of coldest month; Bio7: Temperature annual range; Bio8: Mean temperature of wettest quarter; Bio9: Mean temperature of driest quarter; Bio10: Mean temperature of warmest quarter; Bio11: Mean temperature of coldest quarter; Bio12: Annual precipitation; Bio13: Precipitation of wettest month; Bio14: Precipitation of driest month; Bio15: Precipitation seasonality; Bio16: Precipitation of wettest quarter; Bio17: Precipitation of driest quarter; Bio18: Precipitation of warmest quarter; Bio19: Precipitation of coldest quarter; DEM: Elevation; Wind: Wind speed; Solar: Downward surface shortwave radiation; FVC: Fractional vegetation cover; Dist_water: Distance to water sources. | | | | | | | | |

**Table S3 Descriptive statistics of variable values extracted from domestic poultry outbreak points**

| Variables | Count | Mean | Median | Std | Min | Max | Q25 | Q75 |
| --- | --- | --- | --- | --- | --- | --- | --- | --- |
| Aspect | 111 | 173.4 | 162.9 | 85.6 | 15.8 | 352.9 | 105.4 | 249.8 |
| Bio1 | 111 | 11.3 | 11.7 | 5.2 | -2.6 | 19.6 | 7.3 | 16.2 |
| Bio2 | 111 | 11.8 | 11.6 | 2.2 | 6.7 | 15.2 | 10.6 | 14.1 |
| Bio3 | 111 | 37.7 | 43.8 | 9.8 | 20.9 | 51.2 | 28.0 | 47.2 |
| Bio4 | 111 | 793.6 | 662.9 | 347.1 | 365.4 | 1524.7 | 465.6 | 1095.4 |
| Bio5 | 111 | 26.5 | 26.3 | 5.1 | 11.5 | 39.2 | 23.1 | 30.3 |
| Bio6 | 111 | -6.9 | -10.7 | 8.8 | -22.5 | 6.7 | -14.5 | 2.2 |
| Bio7 | 111 | 33.4 | 32.6 | 9.9 | 20.5 | 52.5 | 23.9 | 42.2 |
| Bio8 | 111 | 19.6 | 20.3 | 4.6 | 6.1 | 29.8 | 16.2 | 22.6 |
| Bio9 | 111 | 2.0 | 0.5 | 8.2 | -13.4 | 15.6 | -4.8 | 9.5 |
| Bio10 | 111 | 20.2 | 20.9 | 4.8 | 6.1 | 30.7 | 16.4 | 23.0 |
| Bio11 | 111 | 0.8 | -1.1 | 8.2 | -13.4 | 14.1 | -5.4 | 9.3 |
| Bio12 | 111 | 581.0 | 419.0 | 397.8 | 26.8 | 1632.8 | 232.2 | 916.0 |
| Bio13 | 111 | 124.6 | 117.8 | 82.5 | 5.5 | 331.8 | 32.5 | 183.9 |
| Bio14 | 111 | 6.4 | 7.0 | 5.2 | 0.0 | 17.0 | 1.0 | 11.2 |
| Bio15 | 111 | 85.3 | 84.8 | 26.8 | 31.2 | 127.3 | 68.8 | 97.0 |
| Bio16 | 111 | 332.0 | 305.0 | 222.2 | 12.0 | 903.8 | 89.5 | 511.4 |
| Bio17 | 111 | 23.2 | 26.8 | 18.7 | 1.5 | 68.0 | 3.4 | 40.8 |
| Bio18 | 111 | 326.0 | 305.0 | 220.1 | 12.0 | 903.8 | 79.4 | 511.4 |
| Bio19 | 111 | 23.5 | 27.0 | 18.8 | 1.5 | 68.5 | 3.4 | 40.8 |
| Chicken | 111 | 755.7 | 179.7 | 1738.4 | 0.0 | 9330.9 | 4.1 | 528.5 |
| DEM | 111 | 1913.5 | 1745.0 | 1209.5 | 35.0 | 4985.0 | 950.0 | 2340.0 |
| Dist_county | 111 | 4.0 | 2.5 | 5.1 | 0.0 | 43.9 | 0.9 | 5.6 |
| Dist_national | 111 | 3.8 | 2.5 | 4.8 | 0.0 | 35.3 | 0.8 | 4.6 |
| Dist_provincial | 111 | 7.8 | 4.4 | 8.5 | 0.2 | 47.3 | 2.2 | 10.0 |
| Dist_railway | 111 | 12.7 | 4.0 | 26.6 | 0.0 | 215.5 | 1.4 | 10.6 |
| Dist_water | 111 | 4.6 | 1.5 | 7.0 | 0.0 | 34.6 | 0.5 | 4.5 |
| Duck | 111 | 5642.9 | 611.2 | 13957.4 | 0.0 | 82041.5 | 6.4 | 4304.4 |
| FVC | 111 | 0.6 | 0.5 | 0.3 | 0.0 | 1.0 | 0.4 | 0.8 |
| GDP | 111 | 28.1 | 33.0 | 21.9 | 0.2 | 57.8 | 0.3 | 47.5 |
| Pop | 111 | 5512.6 | 1402.3 | 10626.7 | 0.0 | 51872.6 | 97.0 | 4600.7 |
| Slope | 111 | 2.6 | 1.3 | 3.3 | 0.0 | 16.7 | 0.5 | 3.0 |
| Solar | 111 | 15309.2 | 15171.3 | 1720.0 | 10549.3 | 17797.1 | 14675.4 | 16269.5 |
| Wild | 111 | 0.3 | 0.1 | 0.3 | 0.0 | 1.0 | 0.0 | 0.6 |
| Wind | 111 | 2.1 | 2.1 | 0.5 | 1.2 | 3.6 | 1.8 | 2.5 |
| **Notes:** Std: standard deviation; Min/Max: the minimum/maximum value; Q25/Q75: 25th and 75th percentiles. Bio1: Annual mean temperature; Bio2: Mean diurnal range; Bio3: Isothermality; Bio4: Temperature seasonality; Bio5: Max temperature of warmest month; Bio6: Min temperature of coldest month; Bio7: Temperature annual range; Bio8: Mean temperature of wettest quarter; Bio9: Mean temperature of driest quarter; Bio10: Mean temperature of warmest quarter; Bio11: Mean temperature of coldest quarter; Bio12: Annual precipitation; Bio13: Precipitation of wettest month; Bio14: Precipitation of driest month; Bio15: Precipitation seasonality; Bio16: Precipitation of wettest quarter; Bio17: Precipitation of driest quarter; Bio18: Precipitation of warmest quarter; Bio19: Precipitation of coldest quarter; GDP: Gross domestic product; Pop: Population density; Dist_railway: Distance to railways; Dist_national: Distance to national highways; Dist_provincial: Distance to provincial highways; Dist_county: Distance to county roads; Chicken: Chicken density; Duck: Duck density; Wild: Risk probabilities of wild birds HPAI; DEM: Elevation; Wind: Wind speed; Solar: Downward surface shortwave radiation; FVC: Fractional vegetation cover; Dist_water: Distance to water sources. | | | | | | | | |

**Table S4 VIFs of the remained variables in three models**

| Variables | Wild | Variables | Domestic | Variables | Mixed |
| --- | --- | --- | --- | --- | --- |
| Bio13 | 7.71 | Bio13 | 7.63 | Bio13 | 7.62 |
| Bio9 | 7.39 | Bio2 | 6.74 | Bio2 | 6.74 |
| Bio2 | 7.06 | Bio14 | 5.71 | Bio14 | 5.68 |
| Bio14 | 6.11 | Bio9 | 5.63 | Bio9 | 5.56 |
| Bio19 | 4.77 | Bio3 | 5.20 | Bio19 | 5.21 |
| Wind | 4.67 | Bio19 | 5.20 | Bio3 | 5.18 |
| FVC | 3.89 | FVC | 4.83 | Wind | 4.58 |
| Bio15 | 2.94 | Wind | 4.60 | FVC | 4.31 |
| Solar | 2.78 | Solar | 3.45 | Solar | 3.30 |
| Dist_water | 1.78 | Bio15 | 3.12 | Bio15 | 3.11 |
| Slope | 1.70 | Dist_national | 2.78 | Dist_national | 2.78 |
| Aspect | 1.01 | Dist_county | 2.57 | Dist_county | 2.57 |
|  |  | Dist_provincial | 2.24 | Dist_provincial | 2.24 |
|  |  | Wild | 2.20 | Dist_railway | 1.97 |
|  |  | Dist_railway | 1.98 | Dist_water | 1.95 |
|  |  | Chicken | 1.70 | Slope | 1.80 |
|  |  | Pop | 1.38 | Chicken | 1.69 |
|  |  | GDP | 1.33 | Pop | 1.43 |
|  |  | Aspect | 1.01 | GDP | 1.38 |
|  |  |  |  | Aspect | 1.01 |
| **Notes:** The Wild, Domestic and Mixed respectively represented the models for wild birds, domestic poultry and the combination of both. VIF: Variance Inflation Factor; Bio13: Precipitation of wettest month; Bio14: Precipitation of driest month; Bio15: Precipitation seasonality; Bio19: Precipitation of coldest quarter; Bio2: Mean diurnal range; Bio3: Isothermality; Bio9: Mean temperature of driest quarter; Chicken: Chicken density; Dist_county: Distance to county roads; Dist_national: Distance to national highways; Dist_provincial: Distance to provincial highways; Dist_railway: Distance to railways; Dist_water: Distance to water sources; FVC: Fractional vegetation cover; GDP: Gross domestic product; Pop: Population density; Solar: Downward surface shortwave radiation; Wild: Simulation results of wild birds HPAI; Wind: Wind speed. | | | | | |

**Fig. S2 Correlation coefficients of the remained variables in three models.** (a) The model for wild birds; (b)The model for domestic poultry; (c) The model for the combination of both. Bio13: Precipitation of wettest month; Bio14: Precipitation of driest month; Bio15: Precipitation seasonality; Bio19: Precipitation of coldest quarter; Bio2: Mean diurnal range; Bio3: Isothermality; Bio9: Mean temperature of driest quarter; Chicken: Chicken density; Dist_county: Distance to county roads; Dist_national: Distance to national highways; Dist_provincial: Distance to provincial highways; Dist_railway: Distance to railways; Dist_water: Distance to water sources; FVC: Fractional vegetation cover; GDP: Gross domestic product; Pop: Population density; Solar: Downward surface shortwave radiation; Wild: Simulation results of wild birds HPAI; Wind: Wind speed.

**Fig. S3 Classification of high- and low-risk areas and hit results of validation points.** Results of the model for (a) wild birds, (b) domestic poultry, and (c) the combination of both.

**Fig. S4 High-risk areas statistics derived from the model for the combination of wild birds and domestic poultry.** YN: Yunnan Province; GS: Gansu Province; XZ: Xizang Autonomous Region; XJ: Xinjiang Uygur Autonomous Region; SC: Sichuan Province; QH: Qinghai Province.

**Fig. S5 Dominant influencing factors and response curves derived from the model for the combination of wild birds and domestic poultry.** (a) Importance of input factors based on permutation importance and percent contribution; (b) Response curves of HPAI risk to dominant factors. Bio15: Precipitation seasonality; Bio19: Precipitation of coldest quarter; Bio2: Mean diurnal range; Bio3: Isothermality; Bio9: Mean temperature of driest quarter; Chicken: Chicken density; Dist_county: Distance to county roads; Dist_national: Distance to national highways; Dist_provincial: Distance to provincial highways; Dist_railway: Distance to railways; Dist_water: Distance to water sources; FVC: Fractional vegetation cover; GDP: Gross domestic product; Pop: Population density; Solar: Downward surface shortwave radiation; Wind: Wind speed.

**Fig. S6. Spatial distribution of dominant factors’ thresholds derived from models.** Results of the model for (a) wild birds, (b) domestic poultry, and (c) the combination of both. Bio19: Precipitation of coldest quarter; Bio9: Mean temperature of driest quarter; Dist_county: Distance to county roads; Dist_national: Distance to national highways; Dist_provincial: Distance to provincial highways; Dist_railway: Distance to railways; Dist_water: Distance to water sources; GDP: Gross domestic product.
